# Deep Convolutional Neural Networks Detect Tumor Genotype from Pathological Tissue Images in Gastrointestinal Stromal Tumors

**DOI:** 10.3390/cancers13225787

**Published:** 2021-11-18

**Authors:** Cher-Wei Liang, Pei-Wei Fang, Hsuan-Ying Huang, Chung-Ming Lo

**Affiliations:** 1 Department of Pathology, Fu Jen Catholic University Hospital, Fu Jen Catholic University, New Taipei City 243, Taiwan; 085027@mail.fju.edu.tw (C.-W.L.); a01156@mail.fjuh.fju.edu.tw (P.-W.F.); 2 School of Medicine, College of Medicine, Fu Jen Catholic University, New Taipei City 242, Taiwan; 3 Graduate Institute of Pathology, College of Medicine, National Taiwan University, Taipei 100, Taiwan; 4 Department of Anatomic Pathology, Chang Gung Memorial Hospital, College of Medicine, Chang Gung University, Kaohsiung 833, Taiwan; hyhuang@cgmh.org.tw; 5 Graduate Institute of Library, Information and Archival Studies, National Chengchi University, Taipei 116, Taiwan

**Keywords:** gastrointestinal stromal tumor, *KIT*, *PDGFRA*, deep convolutional neural network, machine learning

## Abstract

**Simple Summary:**

In this study, we established four convolutional neural network (DCNN) models (AlexNet, ResNet101, DenseNet201, and InceptionV3) to predict drug-sensitive mutations from images of tissues with gastrointestinal stromal tumors. The treatment of these tumors depends on the mutational subtype of the *KIT/PDGFRA* genes. Previous studies rarely focused on mesenchymal tumors and mutational subtypes. More than 5000 images of 365 GISTs from three independent laboratories were used to generate the model. DenseNet201 achieved an accuracy of 87% while the accuracies of AlexNet, InceptionV3, and ResNet101 were 75%, 81%, and 86%, respectively. Cross-institutional inconsistency and the contributions of image color and subcellular components were studied and analyzed.

**Abstract:**

Gastrointestinal stromal tumors (GIST) are common mesenchymal tumors, and their effective treatment depends upon the mutational subtype of the *KIT/PDGFRA* genes. We established deep convolutional neural network (DCNN) models to rapidly predict drug-sensitive mutation subtypes from images of pathological tissue. A total of 5153 pathological images of 365 different GISTs from three different laboratories were collected and divided into training and validation sets. A transfer learning mechanism based on DCNN was used with four different network architectures, to identify cases with drug-sensitive mutations. The accuracy ranged from 87% to 75%. Cross-institutional inconsistency, however, was observed. Using gray-scale images resulted in a 7% drop in accuracy (accuracy 80%, sensitivity 87%, specificity 73%). Using images containing only nuclei (accuracy 81%, sensitivity 87%, specificity 73%) or cytoplasm (accuracy 79%, sensitivity 88%, specificity 67%) produced 6% and 8% drops in accuracy rate, respectively, suggesting buffering effects across subcellular components in DCNN interpretation. The proposed DCNN model successfully inferred cases with drug-sensitive mutations with high accuracy. The contribution of image color and subcellular components was also revealed. These results will help to generate a cheaper and quicker screening method for tumor gene testing.

## 1. Introduction

Mutational analysis of cancer cells is a key, and usually rate-determining, step in targeted therapy. In most circumstances, pathologists diagnose cancers using microscopic examination of histopathology slides, to assess tumor morphology, followed by the selection of representative tumor areas for subsequent nucleic acid extraction and genetic analysis of drug-sensitive mutations. While morphology is the basis for diagnosis, genes are usually the major determinant of treatment. The acquisition of mutational data, however, is usually time consuming, especially in local hospitals where the necessary equipment for gene sequencing is not available. One approach to reducing the time required to diagnose drug-sensitive mutation is the use of deep learning algorithms to provide a suggestion of mutations directly from tumor features. Several studies have addressed this issue of morphology–genotype correlation in cancers [1,2,3,4,5,6]. However, in these studies, researchers primarily focused on identifying the presence of specific mutations within carcinomas, without taking into account the knowledge that, in some circumstances, the mutational subtype, not merely the presence or absence of a mutation, is the main determinant of effective treatment.

In this study gastrointestinal stromal tumors (GISTs) were chosen to be the subject because: (1) GIST is the most common mesenchymal malignancy of the gastrointestinal tract, and reducing the patients’ waiting time has significant clinical impact. (2) The target gene of this tumor, *KIT* or the *PDGFRA* receptor tyrosine kinase gene, has a complex pattern of mutation, which makes morphology–genotype correlation extremely difficult for human perception. (3) Most of the GISTs show consistent morphological features across the whole tumor, making it easy to obtain representative microscopic images. This exempts pathologists from the need for submitting whole-slide images and greatly enhances the efficiency of data transmission. (4) The target gene, *KIT/PDGFRA*, is the major protein-producing gene in GIST, and mutation of this gene is expected to generate considerable morphological variation, which may facilitate detection. (5) Deep learning studies into mesenchymal tumors are rare [2], and mesenchymal tumors arise from an extremely heterogenous group of diseases, with a few cases in each category in open image databases. (6) In GISTs, the subtype of mutations present is more important than the presence of mutation itself. For example, mutations in exon 11 of the *KIT* gene are usually drug-sensitive, while mutations in exon 9 are usually drug-resistant [7]. Genotype–phenotype studies relating mutational subtypes to histology have rarely been performed.

Conventional computer-aided diagnosis systems are based on communication between physicians and computer scientists. Details of the findings useful for diagnosis are passed from physicians to computer scientists, who generate formulas and metrics. Quantitative features are extracted from medical images and analyzed using machine learning algorithms, an approach known as artificial intelligence (AI). Recently, deep convolutional neural networks (DCNNs) have been widely used to automatically generate and combine image features [8] and have successfully improved decision making in areas such as tumor and microorganism detection, evaluation of malignancy, and prediction of prognosis [9,10,11,12]. Instead of using handcrafted features, relevant features can be extracted using multiple convolutional layers. A previous study using a DCNN model achieved an accuracy of 95.3%, a sensitivity of 93.5%, and a specificity of 96.3% for the detection of *Bacillus* in whole-slide pathology images [9]. With this high accuracy, the diagnostic process can be accelerated, and the efficiency enhanced. Quantification of the rich information embedded in histopathology images using DCNNs permits genomic-based correlation and measurement for the prediction of disease prognosis [8].

Furthermore, using multiple convolutional, max pooling, and fully connected layers, the underlying patterns used for classification tasks can be identified and interpreted. Large numbers of features are extracted and combined to represent features ranging from the edges of objects to meaningful interpretations. Using this approach, the manual manipulation of image features can be minimized. One of the requirements for deep learning is the availability of a large number of training images for the neural network. When establishing a model for a new task, the collection of data and training of the network is time consuming. Transfer learning can be helpful in addressing this problem. Especially for medical images, considering the time and cost involved in obtaining data and agreements from patients and the institutional review board, the use of transfer learning to train a target task from a pre-trained model is valuable. This process involves transferring knowledge from numerous natural images from a source such as ImageNet to obtain substantial image features [13,14], and transferring pre-trained weights to the target task to accelerate the learning process.

In this study, GIST histopathology slides were collected from three different laboratories, with images registered by two independent pathologists. A prediction model was constructed using DCNNs. The deep learning system could identify drug-sensitive mutations from tumor cell morphology with an accuracy of more than 85%. Subsequent subcellular studies involving nuclear or cytoplasmic subtraction demonstrated that the cellular alterations were not confined to the nucleus or cytoplasm. We also investigated the impact of the use of color images versus gray-scale images on the accuracy of model prediction. Our study expands the application of AI in daily medical practice.

## 2. Materials and Methods

**Figure 1:**
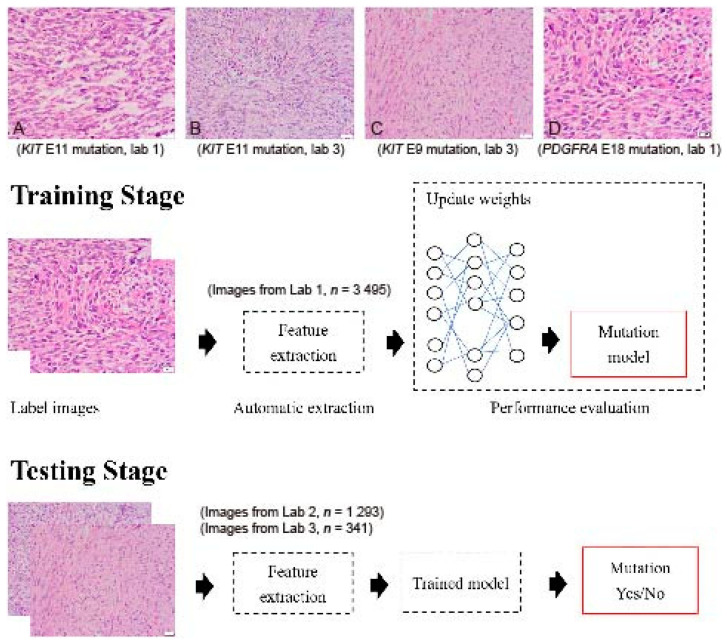
Flowchart of the implemented deep convolutional neural network. Images were collected from 3 independent laboratories (Lab 1, Lab 2, and Lab 3) using 4 µm thick, hematoxylin-and-eosin slides. (**A**) An example image of a GIST with a *KIT* exon 11 mutation from Lab 1 (400×). (**B**) An example image of a GIST with a *KIT* exon 11 mutation from Lab 3 (400×). (**C**) An example image of a GIST with a *KIT* exon 9 mutation from Lab 3 (400×). (**D**) An example image of a GIST with a *PDGFRA* exon 18 mutation from Lab 1 (400×). Although similar staining protocols were used, the overall hue differs between laboratories. A total of 5129 images were enrolled in the study. Four different DCNNs, AlexNet, InceptionV3, ResNet, and DenseNet, were used with transfer learning from ImageNet. The transferred DCNN model generated the probability that each image contained a drug-sensitive mutation. A probability higher than 0.5 was regarded as positive. The dataset was randomly separated into 10 equal-sized subsets. In each iteration, one subset was picked and used to test the trained model on the remaining nine subsets.

GIST histopathology sections, 4 µm thick and stained with hematoxylin and eosin (H&E), were collected from three independent laboratories: Lab 1 (Dr. C.W.L), Lab 2 (Dr. H.Y.H), and Lab 3 (Dr. P.W.F). A total of 5153 H&E sections from 365 tumors were collected, including cases with *KIT* exon 11 mutations (drug-sensitive), *KIT* exon 9 mutations (drug-insensitive), other *KIT* mutations (variable drug sensitivity), *PDGFRA* mutations (variable drug sensitivity), and *KIT/PDGFRA* wild type (WT) mutations (drug-insensitive) (Figure 1A–D). The breakdown of each mutational pattern and the number of cases are listed in Table 1. The overall workflow chart is shown in Figure 1. For each tumor, an average of 15 representative high-power field (400×) images were selected and registered by two pathologists. Areas of necrosis, heavy inflammation, and section artifacts were avoided. Representative images were taken from the tumor region, not including adjacent normal tissue. The images were then uploaded to a Cloud server for subsequent analysis. The study has been approved by the Institute Review Board of Fu Jen Catholic University (No. C107134, approved on 20 June 2019).

Mutational data for each tumor were obtained by Sanger sequencing as previously described [15,16]. The tumors were separated into two groups: the “drug-sensitive” group (*KIT* exon 11 mutations) (Figure 1A,C) and the “other” group (*KIT* exon 9 mutations, *KIT* other mutations, *PDGFRA* mutations, and WT cases) (Figure 1B,D). The aim of this study was to determine whether cases with drug-sensitive *KIT* exon 11 mutations could be predicted by the deep learning system directly from tumor cell morphology.

In the experiment, data from each laboratory were used as a training set (Lab 1), a validation 1 set (Lab 2), and a validation 2 set (Lab 3). Different DCNNs, including AlexNet [17], Inception V3 [18], ResNet101 [19], and DenseNet201 [20] were used with transfer learning, and their performances were evaluated. The parameters trained from ImageNet were transferred into AlexNet, Inception V3, Res-Net101, and DenseNet201 for the task of GIST mutation. Training settings used in MATLAB (MathWorks Inc.) include initial learning rate = 0.001 to achieve the optima weight smoothly, max epochs = 4 as the learning curve is converged, and mini batch size = 64 for the balance between memory and computation. The transferred DCNN model generated the probability that each image contained a drug-sensitive mutation. A probability higher than 0.5 was regarded as positive. To evaluate the generalization ability of the networks, 10-fold cross validation was used [21]. The dataset was randomly separated into 10 equal-sized subsets. In each iteration, one subset was picked and used to test the trained model on the remaining nine subsets. Original color and corresponding gray-scale images were prepared as input sources, as well as images with only cell nuclei and without cell nuclei [2].

## 3. Results

Sample GIST images with different mutations from different laboratories are presented in Figure 1A–D. Although similar staining protocols were used, the images from different laboratories had slightly different color hues.

Table 2 shows the prediction accuracies of the different DCNN models with transfer learning on a combination of all three datasets (70–85%) and of the Lab 1 on the largest dataset. Of the four DCNN models, DenseNet201 produced the best results. Overall, the Lab 1 model established by DenseNet201 provided the best accuracy of prediction (87%). Testing the best Lab 1 DenseNet model on the datasets from the Lab 2 and Lab 3 produced a testing accuracy ranging from 65% to 66%, and the model accuracy trained individually on datasets from the Lab 2 and Lab 3 ranged from 79% to 94% (Table 3). This drop (from 87% to 65% and 66%) and recovery (to 79% and 94%) of accuracies in model cross-applying and processing between different laboratories indicated the presence of cross-institutional inconsistency.

To address this issue and to test which element of the input images had the greatest impact on the prediction accuracy, transformed gray-scale images were prepared to estimate the differentiating power of the staining colors. There was a drop in prediction accuracy from 87% to 80% when input color images were transformed into gray-scale images. Similar approaches were applied to investigate the significance of the cell nuclei and cytoplasm in segregation. There was a drop in prediction accuracy from 87% to 81% when nucleus-only images were input, while a drop in prediction accuracy from 87% to 79% was observed when the nuclei were extracted from the input images. The results are shown in Table 4.

## 4. Discussion

In this study, we aimed to use variable kinds of DCNNs to identify a direct link between tumor cell morphology and the mutational status of specific genes. Several recent studies have addressed a similar issue, that of phenotype–genotype correlation in cancers, using deep learning systems [1,2,3,4,5,6]. However, in these studies, only a few cases of mesenchymal tumors were included [2]. The largest dataset to date, produced by Fu et al. in their 2020 study [2], incorporated 200 malignant mesenchymal tumor (sarcoma) cases. Sarcoma is an extremely heterogenous group of diseases, encompassing more than 50 entities. Although the sarcoma breakdown list was not provided in that study, from the analytical result that *MDM2*, *TP53*, and *RB1* were discriminative genes, one can expect that a significant portion of the dataset comprised liposarcoma and complex karyotype sarcomas such as leiomyosarcoma and osteosarcoma, which are currently not subjects of targeted therapy. Xu et al. conducted a radiogenomic study on GISTs in 2018 and reported that GISTs with and without *KIT* exon 11 mutations could be discriminated by AI texture analysis of enhanced computed tomography images [22]. However, in that study, only four cases of GIST without *KIT* exon 11 mutations were included in the validation cohort, leaving room for discussion of the interpretation [22].

As mentioned in the Introduction, we chose GIST as the subject for several reasons. Cancer tumorigenesis usually involves a stepwise accumulation of hundreds to thousands of genetic mutations. It can be expected that some of these mutations contribute with varying degrees to the morphological changes. Owing to the common intratumoral heterogeneity in cancers, there are usually different regions with different morphologies in the same tumor. Given this diversity, if one wants to establish a link between certain mutations of a gene and certain patterns of morphological changes, thousands of input images may be needed. Submitting whole-slide images for analysis may be beneficial because several morphological patterns can be included simultaneously, and thus exempt pathologists from the need to select representative images of each pattern. However, submitting such whole-slide images generates heavy loads in internet transmission, and the analysis of large amounts of data is time consuming, making this approach less appealing in daily practice. GISTs have the advantages of low mutational burden and low intratumoral heterogeneity, making them suitable subjects for this pioneering study [23,24]. Clinicians can conveniently upload representative .jpg high power field images, which are usually <1000 KB in size, for analysis on a server. Because the drug-sensitive genes (*KIT/PDGFRA*) in GISTs are the major protein-producing genes in these cells, based on their high transcription levels in the gene expression profile [25], they are expected to have a major impact on morphological alterations, and, therefore, the number of input images necessary to reach biological significance may be reduced. The result of our study is encouraging, since, with ~5000 images, a prediction rate with an accuracy of greater than 80% can be obtained.

In the choice of DCNNs for the clinical application, DenseNet201, which performed the best, would be a good one for use. However, heavy computation was needed for the model training and, meanwhile, a large amount of data needed to be collected for the best fitting. These were the two main concerns compared with the conventional image processing and feature extraction methods [26,27]. Nevertheless, automatic feature extraction and combination in DCNN reduced the misunderstanding and time-consuming communications between pathologists and computer scientists, especially for the gene mutation task, where it is difficult to discover sophisticated information from pathological im-ages with human eyes.

In this study, we found cross-institutional inconsistency when applying DCNN models. The cause of this cross-institution reduction in model prediction accuracy may be caused by different slide quality, different staining procedures/reagents, different batch effects (such as humidity and pH value), and varying degrees of slide color fading in storage between different institutions. To explore the cause of cross-institution inconsistency, we assume that the colors in tissue images play an important role in DCNN interpretation, since different laboratories usually have similar but slightly different staining protocols and environments. Pathologists rely on H&E sections to make correct diagnoses. Hematoxylin (H) is the major dye that gives nuclei a blue color, and eosin (E) is the major dye that gives cytoplasm a red color [28]. Within this color spectrum (from deep to light, from blue to red, and its mixture, purple) pathologists gain experience linking certain color combinations to certain diagnoses. By transforming color images to gray-scale images, a drop in prediction rate was observed, indicating that image color did play a role in DCNN interpretation. Paradoxically, an enhancement in the prediction rate was observed when combining all the images for analysis after gray-scale transformation. This process addresses the issue of color inconsistency between different laboratories, which is usually due to different staining protocols and environmental factors, such as different humidity and temperature, may play an even more important role in DCNN interpretation error. The importance of color calibration in input images is therefore clear [29].

As previously mentioned, an importing factor affecting slide staining colors is stain fading in archived and aged samples. Color fading may be asymmetric, uneven, or un-proportionate, and the extent/severity of the fading may be affected by slide storage condition (humidity, temperature, etc.), different staining protocols, quality of the staining reagents, tissue intrinsic factors, and extrinsic artifacts. Most of the studies use de-identified data of which the slide age is not known, as it is in this study. This limitation again stresses the importance of color control in digital imaging, and the United States Food and Drug Administration have released guidance in management of color reproducibility in digital pathology images [30]. We are planning on using more image processing techniques to analyze a future image database with time stamps to clarify this issue.

Surgical pathologists show great interest in knowing the major subcellular locations of morphological changes caused by certain mutations. By segmenting out the nuclei, a significant drop in prediction rate was observed in both nuclei-only and cytoplasm-only images. However, the reduction was limited, leading to the hypothesis that both nuclei and cytoplasm are major subcellular locations of morphological alterations, and combining these two components generates synergic and buffering effects on accurate prediction.

Using state-of-the-art machine learning models keeps shaping our future scope of medical application and can help mitigate technical gaps between different pathology laboratories. In areas where local hospitals cannot afford molecular laboratories, the implementation of prediction models can help doctors obtain tumor mutational data of their patients in minutes, which greatly accelerates the treatment processes. Although the result of this pioneering study is encouraging, there are still challenges. Larger studies with more cases need to be conducted in the future to enhance the prediction accuracy, to expand the classification subcategories, and to include cases with rare mutational patterns. Novel mutations are hardly predicted by machine learning models and doctors should always be aware of cases that are unusual in clinical or pathological presentation.

## 5. Conclusions

In summary, in this study, we demonstrated that a deep learning system can be used to establish a link between cellular morphology and certain gene mutations. This approach helps to reduce the waiting time for mutational analysis, and may benefit the patients by facilitating targeted therapy in the future.

## Figures and Tables

**Table 1:** Breakdown of mutational patterns and number of cases in each data set.

Mutational Pattern	Dataset 1 (Lab 1)		Dataset 2 (Lab 2)		Dataset 3 (Lab 3)		Sum (Case)	Sum (Image)
	Case No.	Image No.	Case No.	Image No.	Case No.	Image No.		
*KIT* E11	125	1959	92	893	15	198	232	3050
*KIT* E9	34	684	2	14	5	104	41	802
*KIT* Non-E9 non-E11	5	130	0	0	1	16	6	146
*PDGFRA*	15	271	6	68	0	0	21	339
*KIT/PDGFRA* WT	29	473	35	320	1	23	65	816
Sum	208	3517 (3495) ^*^	135	1295 (1293) ^*^	22	341 (341) ^*^	365	5153 (5129) ^*^

* Real image numbers entering DCNN calculation after excluding images with artifacts.

**Table 2:** Accuracies of using different DCNNs on combination of three datasets (Lab 1, Lab 2, and Lab 3) and the largest dataset (Lab 1).

DCNN	Combination (Lab 1, Lab2 and Lab 3)	Lab 1
AlexNet	70%	75%
Inception V3	77%	81%
ResNet101	84%	86%
DenseNet201	85%	87%

**Table 3:** Accuracies of the Lab 1 model (DenseNet201) on the Lab 2 and Lab 3 datasets, and model building using the Lab 2 and Lab 3 dataset.

Dataset	Testing Accuracy	Model Accuracy
Lab 2	66%	79%
Lab 3	65%	94%

**Table 4:** Performances of training DenseNet201 on the Lab 1 dataset with gray-scale, only cell nuclei, and without cell nuclei.

Dataset	Accuracy	Sensitivity	Specificity	Positive Predictive Value	Negative Predictive Value	AUC
Gray-scale	80%	87%	73%	79%	83%	0.8645
Only cell nuclei	81%	87%	73%	80%	83%	0.8832
Without cell nuclei	79%	88%	67%	77%	83%	0.8562

## Data Availability

The data presented in this study are available on request from the corresponding author. The data are not publicly available due to ethical restriction on image distribution.
